# Protocol for a systematic review and meta-analysis of cognitive-behavioural therapy for social anxiety disorder in psychosis

**DOI:** 10.1186/2046-4053-3-62

**Published:** 2014-06-11

**Authors:** Maria Michail, Max Birchwood, Lynda Tait

**Affiliations:** 1 School of Health Sciences, Jubilee Campus, University of Nottingham, Nottingham NG7 2TU, UK; 2 Warwick Medical School, University of Warwick, Coventry CV4 7AL, UK; 3 School of Health Sciences, University of Nottingham, Nottingham NG7 2HA, UK

**Keywords:** Systematic review, Meta-analysis, Social anxiety, Psychosis, Schizophrenia, Cognitive-behavioural therapy

## Abstract

**Background:**

Social anxiety is among the most prevalent and debilitating affective disturbances manifest in people with psychosis. It is usually accompanied by high levels of depression and leads to significant social disability, lower quality of life and poorer prognosis as it raises the possibility of an early relapse. Despite its elevated prevalence and severity in psychosis, social anxiety remains under-recognized and under-treated. Cognitive-behavioural therapy is recommended for the treatment of people with psychosis. However, its focus and evaluation has primarily revolved around the reduction of psychotic symptoms, and not for co-morbid affective disturbances such as social anxiety. There is lack of evidence on the clinical effectiveness and cost-effectiveness of cognitive-behavioural interventions for the treatment of social anxiety disorder in psychosis.

**Methods/Design:**

Electronic databases will be systematically searched for randomised controlled trials and quasi-experimental studies investigating the effectiveness and cost-effectiveness of cognitive-behavioural interventions for the treatment of social anxiety disorder in people with psychosis. Grey literature will also be searched by screening trial registers. Only studies published in English will be included in the review. Date restrictions will not be applied. Eligible studies will have as the primary outcome social anxiety (continuous data) measured using any psychometrically validated scale both self-reported and clinician administered. Secondary outcomes will include general anxiety symptoms, distress, depression, positive and negative symptoms of schizophrenia, and quality of life measured using any psychometrically validated scale, both self-reported and clinician administered, and the cost of cognitive-behaviour therapy (CBT) intervention (with another treatment or treatment-as-usual).

**Conclusions:**

This review will provide an evidence synthesis of the effectiveness and cost-effectiveness of cognitive-behavioural interventions for the treatment of social anxiety disorder in people with psychosis. The review will identify the specific intervention components associated with effectiveness which will facilitate the translation of the existing evidence to the development of new, targeted interventions optimising these components. In doing so, this review will provide recommendations for the treatment of social anxiety and associated distress in psychosis and will further inform the development of future interventions in this area.

**Trial registration:**

**PROSPERO registration number**CRD42014009052.

## Background

Social anxiety disorder is among the most frequently reported psychiatric disorders with a lifetime prevalence of 12% and a 12-month prevalence of 7.1% [1]. According to the Diagnostic and Statistical Manual of Mental Disorders, Fifth Edition (DSM-5) [2], social anxiety disorder (previously known as social phobia) is defined as “a marked fear or anxiety about one or more social situations in which the individual is exposed to possible scrutiny by others. Examples include social interactions (e.g., having a conversation), being observed (e.g., eating or drinking), or performing in front of others (e.g., giving a speech).”

People with social anxiety desire to make a favourable impression during social encounters but at the same time doubt their ability to do so; they fear that they will be scrutinized and negatively evaluated due to perceived failed social performance. These fears lead people with social anxiety to avoid all or some social situations and in extreme cases this could lead to complete social isolation [3]. Exposure to the feared situation is almost always accompanied by physical symptoms, for example, sweating, trembling, heart racing, which could develop (although not necessarily) into panic attacks.

Social anxiety develops at an early age, usually during childhood or adolescence and, once established, follows a stable, chronic course if treatment is not initiated ([4,5]; Findings regarding the sociodemographic characteristics of social anxiety disorder support that this is more prominent among females [6-10] although there have been studies [11] which have failed to confirm such gender differences. Higher incident rates have been consistently observed among unmarried individuals usually coming from a lower socioeconomic background, with poorer educational attainment and higher unemployment rates [6,7,10]. The average duration of illness is approximately 29 years [4,12] and the likelihood of a full remission or recovery is significantly lower compared to that of other anxiety disorders [12].

### Social anxiety disorder in psychosis

Social anxiety is among the most prevalent and debilitating affective disturbances manifested in people with psychosis with rates ranging between 8% and 36% ([13-15]; [16,17]; [18,19]). Social anxiety is usually accompanied by high levels of depression ([19];) and leads to significant social disability [17], a reduced quality of life [16] and poorer prognosis as it raises the possibility of an early relapse [20]. Despite its elevated prevalence and severity in psychosis, social anxiety remains under-recognized and under-treated. One of the reasons for this could be that the exact relationship between social anxiety and psychotic symptoms is yet to be determined and available empirical findings remain inconclusive [21]. Although theoretical models and empirical evidence consistently point towards a link between general anxiety and positive symptoms of psychosis, predominantly paranoia and persecutory delusions [22], social anxiety appears to have a distinct quality and its relationship to paranoia and persecutory thinking is not straightforward. Four pathways have been proposed for the understanding of the ontogeny of social anxiety in psychosis [19,23]:

1. Social anxiety predates the onset of paranoia and helps maintain persecutory beliefs

2. Social anxiety and paranoia develop concurrently in the early phase of psychosis and follow a similar course

3. Social anxiety develops as a consequence of paranoid beliefs

4. Social anxiety emerges as a response to the shame and social stigma attached to a diagnosis of mental illness

### Clinical implications

Cognitive-behavioural therapy (CBT) is recommended by the National Institute for Health and Care Excellence [24] for people with psychosis. However, the focus and evaluation of CBT for psychosis has primarily revolved around the reduction of psychotic symptoms, and not for co-morbid depression and social anxiety [23,25]. There is lack of evidence on the clinical effectiveness and cost effectiveness of CBT interventions for the treatment of affective dysregulation and associated distress in psychosis and particularly so for social anxiety disorder.

### Aim

This systematic review and meta-analysis will provide an evidence synthesis of the effectiveness and cost-effectiveness of CBT interventions for the treatment of social anxiety disorder in people with psychosis. In doing so, this review will provide recommendations for the treatment of social anxiety and associated distress in psychosis and will further inform the development of future interventions in this area. To the best of the authors’ knowledge this is the first systematic review of CBT interventions for social anxiety in psychosis.

### Objectives

Our objectives are:

1. To synthesize the evidence of the effectiveness and cost-effectiveness of CBT interventions for the treatment of social anxiety disorder in people with psychosis in improving outcomes related to social anxiety symptoms, general anxiety, distress, depression, positive and negative symptoms of schizophrenia, and quality of life.

2. To estimate the aggregate effectiveness of CBT interventions for the treatment of social anxiety disorder in people with psychosis in improving outcomes related to social anxiety symptoms, general anxiety, distress, depression, positive and negative symptoms of schizophrenia, and quality of life.

## Methods/Design

### Study design

We shall conduct a systematic review and meta-analysis and we will report the outcomes according to the Preferred Reporting Items for Systematic Reviews and Meta-Analyses (PRISMA) statement [26] following the guidelines in the Cochrane Handbook for Systematic Reviews of Interventions [27].

### Search strategy

The literature search strategy is included in Additional file 1. The following search limits will be set: 1) Study design: randomised controlled trials (RCTs) and quasi-experimental studies, 2) limited to English language. Date restrictions will not be applied. The following bibliographic databases will be searched:

▪ Cochrane Central Register of Controlled Trials

▪ CINAHL (Cumulative Index to Nursing and Allied ▪ Health Literature)

▪ EMBASE

▪ MEDLINE

▪ PsychINFO

▪ SCI (Science Citation Index)

### Grey literature

▪ Clinical Trials: clinicaltrials.gov

▪ ISRCTN Register

### Search terms

We shall use Medical Subject Headings (MeSH) and free-text word terms, as appropriate to the databases. The electronic search strategy terms (where necessary using wildcards) are:

Social anxiety

Social phobia

Social anxiety disorder

Schizophrenia

Psychosis

Cognitive therapy

CBT

Effectiveness

Treatment efficacy

Search strategies will be pilot tested. We shall use EndNote to record titles and abstracts for retrieval and inclusion/exclusion decisions.

### Selection criteria

#### Types of participants

Participants will be between 16 and 65 years of age, with schizophrenia or related psychoses (as diagnosed using any recognized diagnostic criteria) with social anxiety disorder as diagnosed using any recognised diagnostic criteria, for example, ICD-10 [28] or DSM-5 [2]. Studies including participants <16 yrs or >65 yrs or participants with a primary diagnosis of organic brain disorder will be excluded from this review.

#### Types of studies

RCTs and quasi-experimental studies will be included in the review.

#### Types of intervention

The review will include cognitive-behavioural (or cognitive) interventions that are targeted at social anxiety in people with psychosis. There will be no limitation in terms of psychological theory informing the intervention, the person delivering the intervention or the setting in which the intervention is delivered. Group and one-to-one interventions will be included.

### Comparator

Control conditions will include any other treatment, no treatment, treatment-as-usual and a waiting list control.

### Types of outcomes

#### Primary outcome

Eligible studies will have as primary outcome social anxiety (continuous data) measured using any psychometrically validated scale, both self-reported and clinician administered.

#### Secondary outcomes

These will include general anxiety symptoms, distress, depression, positive and negative symptoms of schizophrenia and quality of life measured using any psychometrically validated scale both self-reported and clinician administered, and the cost of CBT intervention (with another treatment or treatment-as-usual).

### Selection procedure

Two researchers (MM and LT) will independently screen the title and abstract of retrieved references for inclusion. The full text of all potential eligible studies will be obtained by MM. The next step will involve two researchers (MM and LT) independently assessing obtained references for inclusion. We shall pilot test the procedure on a small number of studies. Any disagreements will be resolved by consensus.

### Managing references

Bibliographic software (EndNote) (Thomson Reuters), will be used to manage retrieved references. MM will be responsible for identifying and removing duplicates, ordering and recording the receipt of any inter-library loans and obtaining the full-text papers. MM will be the sole team member responsible for adding or amending library records in EndNote.

### Data extraction, procedures and data management

The EPOC data extraction form in combination with the EPOC data checklist (see Additional file 2) will be used to extract data from relevant studies. Two reviewers (MM and LT) will work independently to extract data. Disagreements between the two reviewers will be resolved by discussion and consensus, or resolved by a third author (MB). Data extraction will include study setting, study population and participant demographics and baseline characteristics, details of the intervention and control conditions, study methodology, recruitment and study completion rates, outcomes and times of measurement, indicators of acceptability to users, suggested mechanisms of intervention action, information for assessment of the risk of bias and variables related to study quality. Study authors will be contacted to request data missing on methods or results. Information on missing data and dropouts will be assessed for each study. We will report the number of participants included in the final analysis of each study as a proportion of all participants in the study. The possible effects of the missing data will be discussed. Data will be included only for those participants whose results are known.

### Dealing with missing data

Study authors will be contacted to request data missing on methods or results. Information on missing data and dropouts will be assessed for each study. We will report the number of participants included in the final analysis of each study as a proportion of all participants in the study. The possible effects of the missing data will be discussed. Data will be included only for those participants whose results are known.

### Quality assessment

We shall assess the quality of studies and assessment of bias using the Cochrane’s Collaboration tool for assessing risk of bias presented in Additional file 3[29]. Two researchers (MM and LT) will independently rate risk of bias of each study. Discrepancies will be resolved by discussion and consensus.

### Data analysis

#### Measures of treatment effects

We shall summarise and describe the characteristics of the population, interventions and outcomes, using descriptive statistics. Standardised mean differences, Hedges *g,* and weighting studies using inverse of variance will be calculated for continuous outcomes. Risk ratios (RR) will be calculated and we shall use the Mantel-Haenszel method to combine studies. We shall report outcomes using 95% confidence intervals (CI), with random-effects models.

### Assessment of reporting biases

Funnel plots will be drawn to investigate the relationship between study power and effect size. Possible reasons for any asymmetry will be discussed.

### Data synthesis and assessment of heterogeneity

A meta-analysis of RCTs and quasi-experimental studies will be conducted using RevMan.

Statistical tests of heterogeneity (chi-square and I-square) will be carried out. A random-effects model will be used to allow for expected heterogeneity. Effect estimates will be weighted by the inverse of their variance, giving greater weight to larger trials.

Sub group analyses will be carried out for studies with similar research questions based on: a) type of interventions: group-based CBT; individual CBT and b) phase of illness: early onset psychosis vs. chronic.

Sensitivity analysis will be carried out to explore the effects of the addition or removal of lower quality studies.

We will use the GRADE system 11 [30] to assess confidence in the quality of evidence of individual outcomes and strength of recommendations.

## Discussion

CBT is recommended by the National Institute for Health and Care Excellence [24] for people with psychosis. However, its focus and evaluation for psychosis has primarily revolved around the reduction of psychotic symptoms, and not for co-morbid depression and social anxiety [23,25]. There is lack of evidence on the clinical effectiveness and cost effectiveness of CBT interventions for the treatment of affective dysregulation and associated distress in psychosis and particularly so for social anxiety disorder. This review will therefore provide an evidence synthesis of the effectiveness and cost-effectiveness of CBT interventions for the treatment of social anxiety disorder in people with psychosis. The review will identify the specific intervention components associated with effectiveness which will facilitate the translation of the existing evidence to the development of new, targeted interventions optimising these components. In doing so, this review will provide recommendations for the treatment of social anxiety and associated distress in psychosis and will further inform the development of future interventions in this area. To the best of the authors’ knowledge, this is the first systematic review of CBT interventions for social anxiety in psychosis.

## Competing interests

The authors declare that they have no competing interests.

## Authors’ contributions

MM conceived and designed the protocol. LT contributed elements to the design of the protocol. MM and LT jointly wrote the manuscript. All authors (MM, MB and LT) read and approved the final manuscript.

## Supplementary Material

Additional file 1 **Literature search strategy template.** The file provides a description of the literature search strategy. Click here for file

Additional file 2 **Data abstraction form.** Form that will be used by the authors to extract data. Click here for file

Additional file 3 **Quality assessment tool.** Tool that will be used by the authors to assess risk of bias. Click here for file
