# Supplementary material for: Protocol for a systematic review and meta-analysis of cognitive-behavioural therapy for social anxiety disorder in psychosis
Source: Syst Rev. 2014 Jun 11;3:62. doi: 10.1186/2046-4053-3-62 (PMC4065605; doi:10.1186/2046-4053-3-62)
Supplement: Additional file 1 — Literature search strategy template. The file provides a description of the literature search strategy. [file 2046-4053-3-62-S1.pdf]

Literature search strategy template

**Search terms (AND, OR, NOT) and truncation (wildcard characters like *)**	**Social* anxi*<br>OR<br>Social* phob*<br>OR<br>Social anxiety disorder<br><br>AND<br>Schizophreni*<br>Psycho*<br><br>AND<br>Cognitive Therapy<br>OR<br>Cognitive Behavio?r Therapy<br>OR<br>Cognitive Behavio?r Intervention<br>OR<br>CBT<br><br>AND<br>Effective*<br>Efficac***
**Databases searched**	*All relevant databases for the topic area such as: CINAHL, Medline, Proquest Central*<br>Cochrane Central Register of Controlled Trials<br>CINAHL (Cumulative Index to Nursing and Allied Health Literature)<br>EMBASE<br>MEDLINE<br>PsychINFO<br>SCI (Science Citation Index)<br><br>Grey literature:<br>Clinical Trials: clinicaltrials.gov<br><br>ISRCTN Register
**Part of journals searched**	*Did you use: keywords in abstract and title, subject headings and so on?*<br>Keywords in abstract and title<br>MeSH terms
**Years of search**	*This depends on the amount of ongoing research published in journals. E.g. 2001-2011*<br>No date restrictions will be applied

**Language**	*E.g. English*<br><br>English language only
**Types of studies to be included**	*E.g. qualitative studies*<br>Randomised-controlled trials (RCTs)<br>Quasi-experimental studies
**Inclusion criteria (why did you include it?)**	***Types of participants***<br>**Inclusion criteria**<br>16-65 yrs<br>With schizophrenia or related psychosis (as diagnosed using any recognised diagnostic criteria e.g. ICD-10; DSM-V)<br>With social anxiety disorder (as diagnosed using any recognised diagnostic criteria e.g. ICD-10; DSM-V)<br><br>Exclusion criteria<br><16 yrs or >65yrs<br>Primary diagnosis of organic brain disorder<br><br>***Types of trials***<br>Randomised controlled trials and quasi-experimental designs.<br><br>***Types of interventions***<br>Cognitive Behavioural Therapy<br>Cognitive Therapy<br>Cognitive Behavioural<br>CBT<br>Both group-based and individual<br>***Control conditions***<br>Control conditions will include any other intervention, no intervention/usual care and waiting list<br>***Types of Outcomes***<br>Primary Outcome: Social anxiety symptoms<br>Secondary Outcomes: General anxiety; distress; depression; positive and negative symptoms of schizophrenia ; quality of life; cost of CBT intervention
**Exclusion criteria (why did you rule it out?**	Systematic reviews, dissertations/theses or studies describing a CBT intervention but not providing any evaluation will be excluded.
