# Supplementary material for: Protocol for a systematic review and meta-analysis of cognitive-behavioural therapy for social anxiety disorder in psychosis
Source: Syst Rev. 2014 Jun 11;3:62. doi: 10.1186/2046-4053-3-62 (PMC4065605; doi:10.1186/2046-4053-3-62)
Supplement: Additional file 2 — Data abstraction form. Form that will be used by the authors to extract data. [file 2046-4053-3-62-S2.pdf]

Cochrane Effective Practice and Organisation of Care Group (EPOC)[1]

# Data Abstraction Form

This form can be used to record the results of data extraction and is intended for use in conjunction with the EPOC Data Collection Checklist.

Data collection

| |
|---|
| Name of reviewer: |
| Date: |
| Study reference: |

EPOC scope:

The effect(s) of a behavioural/educational, financial, organisational or regulatory intervention(s) is evaluated

## 1. Inclusion criteria

### 1.1 Study design

#### 1.1.1 RCT designs

#### 1.1.2 CCT designs

#### 1.1.3 CBA designs

a) Contemporaneous data collection

b) Appropriate choice of control site/activity

- Studies should contain a minimum of two intervention and two control groups

#### 1.1.4 ITS designs

a) Clearly defined point in time when the intervention occurred

---

[1]EPOC Editorial Base:

Alain Mayhew, Managing Editor
Cochrane Effective Practice and Organisation of Care Group
Centre for Practice-Changing Research, Ottawa Hospital Research Institute
501 Smyth Avenue, Box 711
Ottawa, Ontario K1H 8L6
Tel: +1 613 737 8899 ext. 73849
Fax: +1 613 739 6938
Email: almayhew@ohri.ca

b) At least 3 data points before and 3 after the intervention

1.2 Methodological inclusion criteria

a) The objective measurement of performance/provider behaviour or health/patient outcomes

b) Relevant and interpretable data presented or obtainable

*N.B. A study must meet the minimum criteria for EPOC scope, design, and methodology for inclusion in EPOC reviews. If it does not, COLLECT NO FURTHER DATA.*

## 2. Interventions

2.1 Type of intervention
(state all interventions for each comparison/study group)

Group 1:

Group 2:

Group 3:

Group 4:

2.2 Control(s)

## 3. Type of Targeted Behaviour (state more than one where appropriate)

## 4. Participants

4.1 Characteristics of participating providers

4.1.1 Profession

4.1.2 Level of training

4.1.3 Clinical specialty

4.1.4 Age

4.1.5 Time since graduation (or years in practice)

4.2 Characteristics of Participating patients

4.2.1 Clinical problem

4.2.2 Other patient characteristics

a) Age

b) Gender

c) Ethnicity

d) Other (specify)

4.2.3 Number of patients included in the study

a) Episodes of care

b) Patients

c) Providers

d) Practices

e) Hospitals

f) Communities or regions

## 5. Setting

5.1 Reimbursement system

5.2 Location of Care

5.3 Academic status

5.4 Country

5.5 Proportion of eligible providers (or allocation units)

## 6. Methods

### 6.1 Unit of allocation

### 6.2 Unit of analysis

### 6.3 Power calculation

### 6.4 Quality criteria

#### 6.4.1 Quality criteria for randomised controlled trials (RCTs) and controlled clinical trials (CCTs)

(Go to 6.4.2 and 6.4.3 for the quality criteria for CBA and ITS, respectively)

a) Concealment of allocation

b) Follow-up of professionals

c) Follow-up of patients or episodes of care

d) Blinded assessment of primary outcome(s)

e) Baseline measurement

f) Reliable primary outcome measure(s)

g) Protection against contamination

#### 6.4.2 Quality criteria for controlled before and after (CBA) designs

a) Baseline measurement

b) Characteristics for studies using second site as control

c) Blinded assessment of primary outcome(s)* (protection against detection bias)

d) Protection against contamination (studies using second site as control)

e) Reliable primary outcome measure(s)

f) Follow-up of professionals (protection against exclusion bias)

g) Follow-up of patients

#### 6.4.3 Quality criteria for interrupted time series (ITS) designs

Protection against secular changes:

a) The intervention is independent of other changes

b) Data were analysed appropriately

c) Reason for the number of points pre- and post-intervention given

d) Shape of the intervention effect was specified

Protection against detection bias:

a) Intervention unlikely to affect data collection

b) Blinded assessment of primary outcome(s)

c) Completeness of data set

d) Reliable primary outcome measure(s)

#### 6.4.4 Consumer involvement

## 7. Prospective identification by investigators of barriers to change

## 8. Intervention

### 8.1 Characteristics of the intervention

a) Evidence base of recommendation

b) Purpose of recommendations

8.2 Nature of desired change

8.3 Format (Medium for each intervention)

8.4 Source

8.5 Intervention based upon implementation of clinical practice guidelines

8.6 Clinical practice guidelines developed through formal consensus process

8.7 Recipient

8.8 Deliverer

8.9 Timing

a) Proximity to clinical decision-making

b) Frequency/number of intervention events

c) Duration of intervention

8.10 Setting of intervention

8.11 Source of funding

8.12 Ethical approval

## 9. Outcomes

9.1 Description of the main outcome measure(s).

a) Health professional outcomes/process measures

b) Patient outcomes

c) Economic variables

- Costs of the intervention
- Changes in direct health care costs as a result of the intervention
- Changes in non-health care costs as a result of the intervention
- Costs associated with the intervention are linked with provider or patient outcomes in an economic evaluation

9.2 Length of time during which outcomes were measured after initiation of the intervention.

9.3 Length of post- intervention follow-up period.

9.4 Identify a possible ceiling effect:

a) Identified by investigator

b) Identified by reviewer

## 10. Results (use extra page if necessary)

10.1.1 For RCTs and CCTs

10.1.2 For CBAs

10.1.3 For ITSs
