# Supplementary material for: Protocol for a systematic review and meta-analysis of cognitive-behavioural therapy for social anxiety disorder in psychosis
Source: Syst Rev. 2014 Jun 11;3:62. doi: 10.1186/2046-4053-3-62 (PMC4065605; doi:10.1186/2046-4053-3-62)
Supplement: Additional file 3 — Quality assessment tool. Tool that will be used by the authors to assess risk of bias. [file 2046-4053-3-62-S3.pdf]

Appendix C Quality Assessment Tool

Table 1| Cochrane Collaboration's tool for assessing risk of bias (adapted from Higgins and Altman13)

Bias domain	Source of bias	Support for judgment	Review authors' judgment (assess as low, unclear or high risk of bias)
Selection bias	Random sequence generation	Describe the method used to generate the allocation sequence in sufficient detail to allow an assessment of whether it should produce comparable groups	Selection bias (biased allocation to interventions) due to inadequate generation of a randomised sequence
	Allocation concealment	Describe the method used to conceal the allocation sequence in sufficient detail to determine whether intervention allocations could have been foreseen before or during enrolment	Selection bias (biased allocation to interventions) due to inadequate concealment of allocations before assignment
Performance bias	Blinding of participants and personnel*	Describe all measures used, if any, to blind trial participants and researchers from knowledge of which intervention a participant received. Provide any information relating to whether the intended blinding was effective	Performance bias due to knowledge of the allocated interventions by participants and personnel during the study
Detection bias	Blinding of outcome assessment*	Describe all measures used, if any, to blind outcome assessment from knowledge of which intervention a participant received. Provide any information relating to whether the intended blinding was effective	Detection bias due to knowledge of the allocated interventions by outcome assessment
Attrition bias	Incomplete outcome data*	Describe the completeness of outcome data for each main outcome, including attrition and exclusions from the analysis. State whether attrition and exclusions were reported, the numbers in each intervention group (compared with total randomised participants), reasons for attrition or exclusions where reported, and any reinclusions in analyses for the review	Attrition bias due to amount, nature, or handling of incomplete outcome data
Reporting bias	Selective reporting	State how selective outcome reporting was examined and what was found	Reporting bias due to selective outcome reporting
Other bias	Anything else, ideally prespecified	State any important concerns about bias not covered in the other domains in the tool	Bias due to problems not covered elsewhere

*Assessments should be made for each main outcome or class of outcomes.
